# ROS-Based Autonomous Navigation Robot Platform with Stepping Motor

**DOI:** 10.3390/s23073648

**Published:** 2023-03-31

**Authors:** Shengmin Zhao, Seung-Hoon Hwang

**Affiliations:** Division of Electronics and Electrical Engineering, Dongguk University, Seoul 04620, Republic of Korea; shengmin.zhao@dgu.ac.kr

**Keywords:** robot operating system, indoor navigation robot, stepping motor, simultaneous localisation and mapping, autonomous navigation

## Abstract

Indoor navigation robots, which have been developed using a robot operating system, typically use a direct current motor as a motion actuator. Their control algorithm is generally complex and requires the cooperation of sensors such as wheel encoders to correct errors. For this study, an autonomous navigation robot platform named Owlbot was designed, which is equipped with a stepping motor as a mobile actuator. In addition, a stepping motor control algorithm was developed using polynomial equations, which can effectively convert speed instructions to generate control signals for accurately operating the motor. Using 2D LiDAR and an inertial measurement unit as the primary sensors, simultaneous localization, mapping, and autonomous navigation are realised based on the particle filtering mapping algorithm. The experimental results show that Owlbot can effectively map the unknown environment and realise autonomous navigation through the proposed control algorithm, with a maximum movement error being smaller than 0.015 m.

## 1. Introduction

As an essential part of Industry 4.0 [1], mobile robots [2] have undergone rapid advancements to exhibit high speed, high precision, openness, and intelligence. Various types of mobile robots have been developed for applications such as path-following autonomous underwater vehicles [3], manipulators based on supertwisting zeroing neural networks [4], and quadrotor helicopters [5]. Notably, most mobile robots have been developed based on the robot operating system (ROS), which is an open-source and flexible framework for writing robotics software [6]. In particular, the ROS is a modular software platform for developing complex robotic applications [7], which can be used to establish models of complex robots and simulate and control robots [8]. Robots can be integrated with simulation and visualisation tools [9,10], libraries for robot vision, simultaneous localization and mapping (SLAM) [11], and navigation [12].

According to the control system components, a mobile robot platform must be designed considering the controller, sensor, and actuator with driver. As core hardware control systems, single-board computers (SBCs) [13] such as Raspberry Pi [14] and Jetson Nano [15] exhibit various capabilities such as joint control, human–computer interaction, and algorithm processing. When combined with PC control, the robot can realise remote monitoring, graphic display, path planning, and other functions. In general, sensors can be divided into two categories depending on the measurement target: sensors that measure robot states, such as the robot position or velocity, and sensors that measure the environmental state. Robotic state-measuring sensors include the Global Positioning System (GPS) [16], inertial measurement units (IMUs) [17], and encoders [18]. IMUs and environmental sensors [19], such as millimetre-wave radars [20] and cameras, can be integrated to allow the robot collect real-time information regarding moving objects or obstacles in the environment to prevent collisions.

When designing a robot platform, indoor mobile robots typically use a DC motor as the actuator and a pulse-width modulation (PWM) driver board as the driver [21]. For the engine to respond, the motor must provide the necessary angular speed and torque in the range of the motor parameters. The motor rotation and direction for an ROS-based mobile robot must be controlled. To this end, power-switching transistors are often used to build H-bridge circuits. In this context, traditional algorithms such as the proportional integral derivative algorithm has been widely used to control the DC motor with the feedback loop mechanism [22]. Traditional DC motor control algorithms control the motor speed with a continuous voltage and implement the next step based on feedback from the motor. Consequently, ROS-based robots with DC motors exhibit low operational accuracy. To continuously correct the position, the control algorithms must operate in tandem with an IMU, encoder, or other sensors, resulting in the high complexity of DC motor control. To avoid this problem, a stepping motor has been used as the robot actuator [23]. A stepping motor converts electrical pulse signals into corresponding angular or linear displacements. For each input pulse, the rotor turns by a specific angle. Therefore, the resultant angular or linear displacement is proportional to the number of input pulses. In addition, the rotational speed is proportional to the pulse frequency. Because the stepping motor can maintain operation within a given step, it can ensure high precision. In this manner, the stepping motor can enable simple, reliable, and real-time control to ensure the accurate movement and positioning of the mobile robot.

Considering these aspects, this paper proposes an autonomous navigation robot platform named Owlbot, which uses a stepping motor as a mobile actuator. Additionally, a stepping motor control algorithm was developed that can effectively convert the speed instructions to generate control signals for ensuring accurate motor operation. The performance of the proposed methods is evaluated through experiments in which SLAM and autonomous navigation are realised based on the particle filtering mapping algorithm using two-dimensional (2D) LiDAR and an IMU as the primary sensors. The contributions of this research can be summarised as follows. First, we designed a novel robot platform named Owlbot with the open-source software platform of ROS. Owlbot consists of hardware components such as a variety of environmental sensors, high-performance SBCs, and stepping motors. Second, a novel stepping motor control algorithm is proposed, which allows the robot to move with high precision. Furthermore, SLAM and autonomous navigation were realised by the fusion of data of various sensors.

This paper is organised as follows: Section 2 summarises previous works. Section 3 describes the physical structure design and software of Owlbot and its hardware components. Section 4 introduces the proposed stepping motor control algorithm. Section 5 discusses the SLAM and navigation performance evaluation of the Owlbot robotic platform. Section 6 presents the concluding remarks.

## 2. Previous Work

Table 1 introduces the software and hardware components (e.g., controller, actuator, and sensors) of well-known mobile robot platforms and the proposed Owlbot robot. The last column specifies the main functions of the robot platforms.

MBot [24] is an entry-level educational robot developed by Makeblock. MBot has a modular design to ensure that it can be connected to various ordinary sensors and programmed through the Arduino IDE. Tiny:bit [25] was designed based on the Micro:bit development board, using a graphical module and Python for programming. This robot is lightweight, easy to assemble, and can move easily in tight spaces. Unlike MBot, Tiny:bit is equipped with a sound sensor and an infrared proximity sensor. The G1 tank [26] robot uses the Raspberry Pi board as the core controller and the expansion board as the driver. This robot can realise various recognition functions, such as object recognition, using its onboard camera. Notably, MBot and Tiny:bit use self-designed integrated motherboards as the controllers and plastic DC gearbox motors [33] as the actuators. Owing to the limited performance of such hardware, only simple mobile functions can be realised, such as line inspection and detection. Although the G1 robot is equipped with Raspberry Pi and DC motors, it does not support the ROS system, and thus, its application scenarios are limited.

In comparison, mobile robots using Raspberry Pi or Jetson Nano as controllers can realise more complex tasks with the help of ROS and additional sensors. Jetbot Mini [27] is an ROS artificial intelligence robot equipped with a Jetson Nano unit and camera. Similarly, the Jetbot project [28] is an open-source intelligent car project based on NVIDIA Jetson Nano. These robots can realise obstacle avoidance, line patrolling, and object recognition. Transbot [29], which uses a depth camera sensor and radar, has functionalities such as remote control capabilities, map navigation, automatic driving, and manipulator operation. Turtlebot 3 [30] is a low-cost, small robotic mobility platform based on the ROS. Leo Rover [31] is a giant robotic platform that adopts four independent DC gear motors with suspension as the actuators and can be used in outdoor environments. The Summit-XL platform from Robotnik [32] is a versatile and robust robot frame designed for high load capacities. This robot is equipped with an IMU, cameras, laser scanners, and a radio system for remote operation.

Notably, the actuator systems of the abovementioned robots are DC motors and encoders. The encoder acts as a position sensor for the DC motor, and the wheel odometer data are calculated from the previous position. Therefore, if a pose estimation error exists in each step, the error will accumulate as the robot moves. To reduce such errors, the robotic platforms typically fuse IMU and encoder data. Traditional DC motor control algorithms control motor speed through continuous voltage and implement the next step based on feedback from the motor. In contrast, the stepping motor control algorithm proposed in this study controls motor speed by the number of pulses and time delays, thereby ensuring the timely control of the robot’s motion, which is crucial for smart factory applications. Furthermore, the stepping motor can be scheduled for runs according to the pulse information, rendering it valuable for special applications such as depth-gauging robots. The stepping motor can respond immediately to the speed command from the ROS navigation node with the help of the control algorithm. Compared with IMU and encoder-based solutions, Owlbot can achieve better performance for SLAM and autonomous navigation in indoor environments.

## 3. Proposed Robot Architecture

### 3.1. Hardware

**Figure 1:**
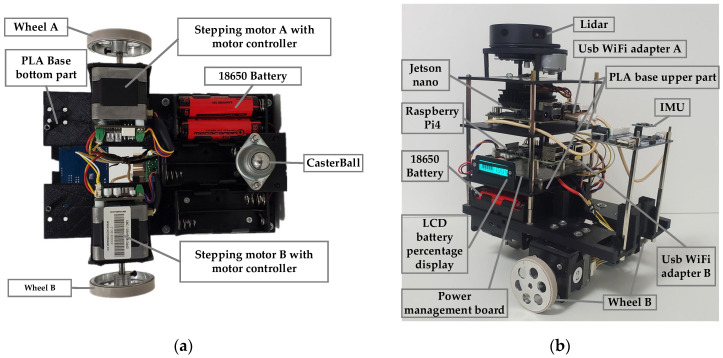
Owlbot robot and its components. (**a**) Bottom view; (**b**) Side view.

**Figure 2:**
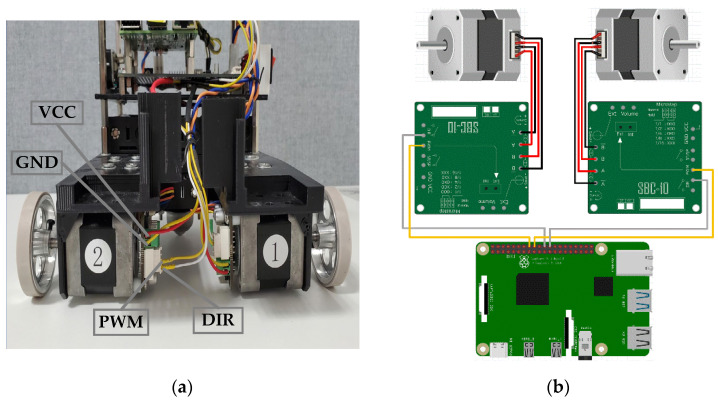
Motor connections of Owlbot. (**a**) Installation diagram; (**b**) Connection between the stepping motor, SBC-10 controller, and Raspberry Pi 4B.

The Owlbot platform is an inexpensive, two-wheel differential drive robot. The platform is sufficiently expandable and highly stable and can carry different sensors such as LiDARs and IMUs. The Owlbot robot is open and self-designed; thus, it can be adapted to different applications through modifications and improvements. Figure 1 shows the robot setup. The Owlbot robot can move with high precision owing to the use of two high-precision stepping motors and wheels. The robot has a Raspberry Pi unit to subscribe to the ROS speed command and connect with the motor controller to enable precise control of the stepping motor. Moreover, the Jetson Nano functions as the robot’s brain and performs information processing, such as the fusion of sensor information, path planning, and communication with the remote control terminal. As essential components of Owlbot, a radar and an IMU facilitate a series of tasks such as map construction and navigation. Furthermore, the robot platform uses a self-designed power management motherboard that can simultaneously provide adequate power for all electronic devices of the robot. The bottom part of Owlbot is a polylactide base fabricated through 3D printing to support various physical and electronic devices. The base is equipped with six groups of 18650 lithium battery bases. As shown in Figure 2, Owlbot moves using two high-precision stepping motors installed with a motor control board. The stepping motor controllers are microprocessor-embedded, voltage-controlled, and miniature stepping motor controllers. Specifically, SBC-10 is a sub-miniature controller with a standard DIP18 land pattern (0.6′ width), and it supports a voltage of 8–28 DC.

**Figure 3:**
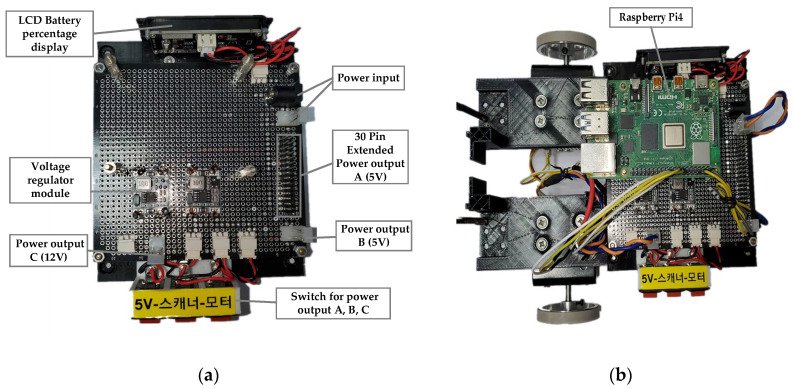
First layer of Owlbot. (**a**) Power management board; (**b**) Installation diagram.

In the top part, Owlbot has three layers. The first layer incorporates a power management board that can simultaneously output different voltages to satisfy the power requirements of various electronic devices. As shown in Figure 3, the power management board can simultaneously output 5 V and 12 V to supply power to the SBC and stepping motor, respectively. The Raspberry Pi unit is connected to the stepping motor driver board, which uses PWM software to generate a PWM signal to drive the stepping motor. The second layer houses the Jetson Nano board, which enables information processing, such as secure shell (SSH) communication with the Raspberry Pi and motion planning. A laser scanner (RPLIDAR A1; Slamtec) for distance measurement is installed in the topmost layer. This laser scanner operates on the principle of laser triangulation and adopts high-speed vision acquisition and processing hardware. The system measures the distance data more than 8000 times per second. The robot is equipped with an IMU at the front end, the data of which can be integrated with the laser data during navigation to achieve precise positioning. Table 2 presents the main hardware composition of the robot platform.

### 3.2. Software

**Figure 4:**
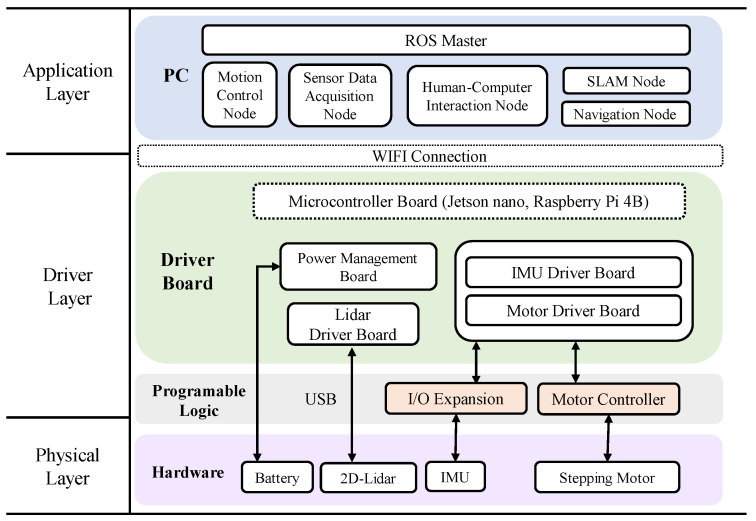
System structure of the Owlbot robotic platform.

The Owlbot software system is based on the distributed framework of the ROS. Owlbot uses the ROS infrastructure for communication and control, as shown in Figure 4. Owlbot contains a physical layer, a driver layer, and an application layer. The physical layer contains hardware such as the battery, 2D LiDAR, IMU, and stepping motor. LiDAR can acquire abundant information, including 2D data, compared with that obtained using a traditional laser or camera. Owlbot uses LiDAR and IMU to determine the position and angle. The driver layer is divided into the programmable logic and processor system. In addition to the programmable hardware, the I/O expansion and motor controller are the main programmable logic characteristics of this layer. The other drivers serve as interfaces to the processor system and device drivers for the operating system. The ROS core library is ported to the embedded Linux operating system (Ubuntu) based on Jetson Nano and Raspberry Pi to exploit the ROS communication mechanism. The system development is based on the ROS interfaces of the robotics middleware with Python. Owing to the excellent design of ROS, the parameters can be conveniently reconfigured online without recompiling the source codes.

The ROS master is used to manage all robot nodes, which can be visualised through a visual component named Robot Visualizer (Rviz). The ROS master runs on the PC. Communication between the ROS master and microcontroller board is accomplished through 802.11n networking. When the Wi-Fi boots up, it creates a hotspot. The SSH can be integrated with Raspberry Pi, and the robot can be directly controlled from a laptop. The master realises a standard function such as SLAM or navigation. The commonly used SLAM algorithms for ROSs are Hector slam [34], gmapping [35], and Karto slam [36].

**Figure 5:**
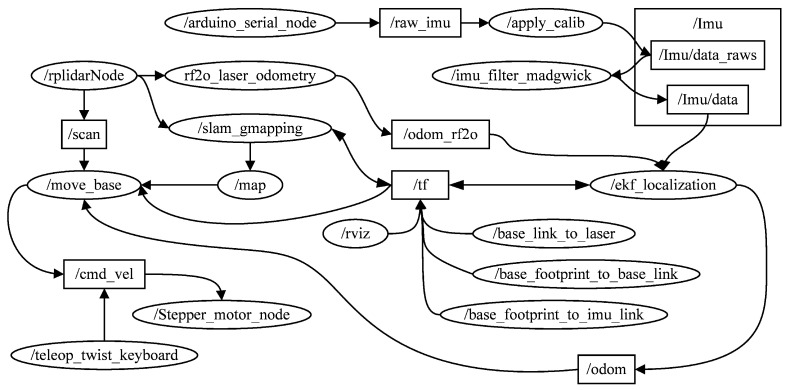
ROS topics and nodes associated with SLAM.

**Figure 6:**
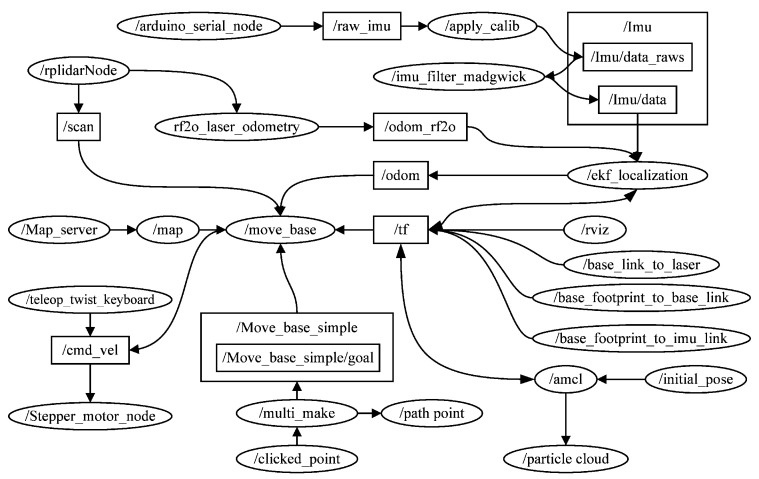
ROS topics and nodes associated with navigation.

The ROS system consists of multiple independent nodes, each of which communicates with other nodes through publish/subscribe messaging. Figure 5 and Figure 6 illustrate the realisation of SLAM and navigation, respectively, with the corresponding ROS topics and nodes. Ellipses represent nodes, squares represent the topics, and arrows represent the message flow. Nodes, as executable entities, can communicate with one another. A complete robot control system consists of many nodes responsible for different functions. For example, the node/RplidarNode in Figure 5 is responsible for controlling the LiDAR publishing topics/scan/rf2o_laser_odometry, and/slam_gamapping. The topic is a name used to identify messages to which nodes can publish messages. Different nodes can subscribe to different topics to receive messages. Node: /move_base obtains 2D LiDAR information by subscribing to the topic/scan.

The Owlbot robot uses the gmapping method for SLAM. This particle filter-based algorithm represents the posterior probability of a path through many weighted particles, each of which is assigned an essential factor. Owlbot has a high-precision stepping motor. To ensure highly accurate operation, the robot additionally employs a LiDAR odometer to fuse measurements. Most of the existing robots typically have DC motors and wheel encoders to output the odometer information/odom. In contrast, Owlbot uses a stepping motor without an encoder. Although the stepping motor runs accurately, we added a 2D LiDAR odometer node (rf2o_laser_odometry) to address inaccurate output odometer data such as wheel suspension and skid. The rf2o_laser_odometry (RF2O) [37] module performs the matching of two adjacent frames of LiDAR data to obtain the mileage displacement. In general, RF2O is a fast and precise method for estimating the planar motion of LiDARs from successive range scans. For each scan point, a distance flow constraint equation is formulated based on the sensor velocity, and a robust function of the resulting geometric constraints is minimised to obtain a motion estimate. Furthermore, the pose is estimated through the fusion of the LiDAR odometer information and IMU information by the extended Kalman filter (EKF). Specifically, the EKF is used to estimate the 3D pose of a robot, based on (partial) pose measurements from different sources [38]. The key concept is to ensure loosely coupled integration with different sensors, with the sensor signals received as ROS messages. The/ekf_localization node uses the relative pose differences of each sensor to update the EKF for pose interpretation.

In the navigation process, the main node is move_base, which pertains to the participation of the navigation control framework for robot path planning. This move_base node subscribes to topics such as/scan,/map, and/odom, and then publishes the speed command (/cmd_vel). The stepping motor control algorithm described in Section 4 is used to drive the motor to run according to the speed command. The control algorithm node is/stepper_motor_node. The stepping motor control algorithm subscribes to the command/cmd_vel from/move_base, converts it into the pulse number and delay required by the motor, and finally drives the motor.

## 4. Proposed Stepping Motor Control Algorithm

Owlbot uses a double-stepping motor as a moving driver. The basic parameters of the motor are listed in Table 3. The motor is an open-loop control element stepping motor that converts electrical pulse signals into angular or linear displacements. With each input pulse, the motor rotates by a certain angle, also known as the step angle. The number of step angles that the motor rotates by is proportional to the number of input pulses, and the speed is proportional to the pulse frequency.

The wheel size considerably affects a robot’s speed. For example, a robot with large wheels will move a longer distance once its motor rotates. Furthermore, after the motor receives the ROS topic/cmd_vel, it must be appropriately converted to the pulse number and frequency. To consider these aspects, we propose a stepping motor control algorithm based on a polynomial regression equation. Table 4 lists the variables involved in the algorithm and their descriptions. The left and right motors (*LM, RM*) have wheels (*LW, RW*) with the same radius ($W_{r}$). The stepping motors use a 1/4 microstep (${LM}_{mic}$*,* ${RM}_{mic}$). Specifically, if 800 PWM pulses are provided, the stepping motor will perform one revolution with a rotation angle of 360°.

Notably, under actual ROS control, the speed command/cmd_vel is issued frequently, which may result in stalling of the stepping motor. A rotating magnetic field is present inside the stepping motor. When the rotating magnetic field is sequentially switched, the rotor rotates. However, when the magnetic field rotates excessively fast or the moment of inertia of the load on the rotor is extremely large, the rotor may not be able to maintain its rhythm, resulting in loss of step. Therefore, the release frequency of cmd_vel must be carefully considered. If the speed release frequency is too small, the movement speed requirements of the robot platform may not be satisfied. In contrast, if the frequency is too high, the motor will be blocked. Therefore, we set the publish and subscription frequencies of cmd_vel as 1 Hz. Considering the movement of the robot in the indoor environment, we set the linear velocity range of Owlbot as −0.09 m/s to 0.09 m/s and the acceleration range as −0.03 $m/s^{2}$ to 0.03 $m/s^{2}$.

**Figure 7:**
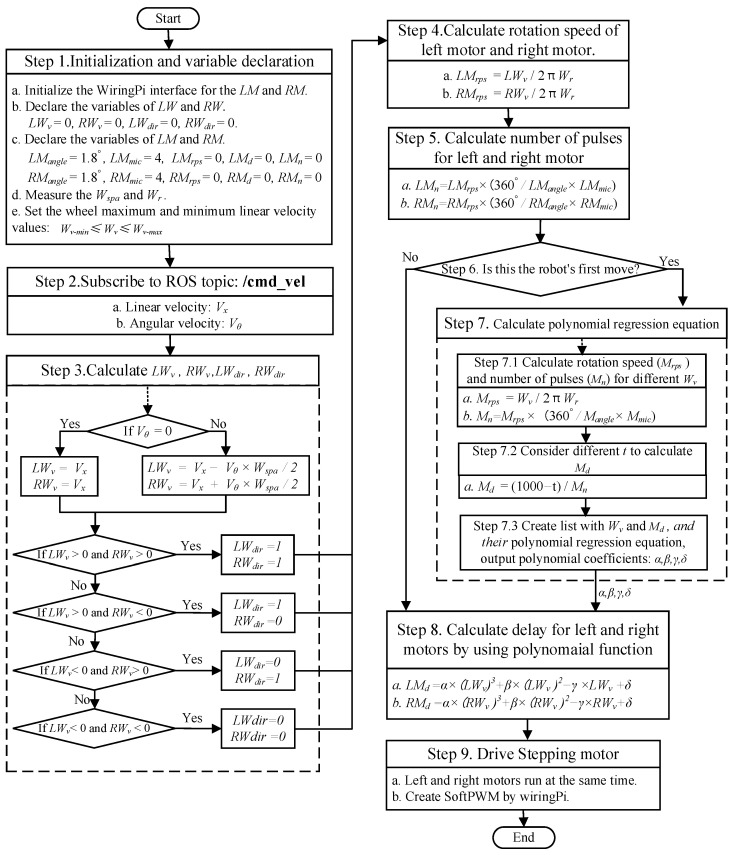
Process flow of the proposed stepping motor control algorithm based on the polynomial regression equation.

Figure 7 shows the process flow of the proposed control algorithm, and its pseudocode is presented as Algorithm 1. The algorithm involves the following steps:

- Step 1: Initialization and variable declaration: We use Raspberry Pi 4B as the control board of the stepping motor. After connecting the Raspberry Pi and the stepping motor, the WiringPI interface is initialised. Initial values are assigned to the relevant parameters of the left and right motors and wheels.
- Step 2: Subscribe to ROS topics:/cmd_vel. Obtain the linear velocity ($V_{x}$) and angular velocity ($V_{\theta }$) that the robot needs to achieve.
- Step 3: Calculate the motor velocity and direction. Convert the speed command (/cmd_vel) into the left wheel velocity (${LW}_{v}$) and right wheel velocity (${RW}_{v}$). Use the judgement condition to define whether the motor rotates clockwise or counterclockwise. A value of 1 represents clockwise rotation.
- Step 4: Calculate the rotation speed (${LM}_{rps}$, ${RM}_{rps}$) of the left and right motors. Because the frequency of the speed instruction is 1 Hz, in this study, we consider the instantaneous rotation speed of the wheel at a point in revolutions per second. Using the formula for rotation speed shown in Figure 7, we can easily convert the speed of the wheel into the rotation speed of the motor.
- Step 5: Calculate the number of pulses for the left and right motors. Based on the calculation in the previous step, we have the required ${LM}_{rps}$ and ${RM}_{rps}$ for the left and right motors. Therefore, we calculate the number of pulses (${LM}_{n}$, ${RM}_{n}$) required to achieve the specified values (${LM}_{rps}$, ${RM}_{rps}$).
- Step 6: Determine whether the robot is moving for the first time. For the first move, the robot must calculate and output the coefficients of polynomial equations. If the robot has already started moving, the polynomial equation does not need to be recalculated.
- Step 7: Solve the polynomial regression equation to obtain the fitting curve of the wheel speed ($W_{v}$) and PWM time delay ($M_{d}$), that is, a polynomial equation.
  o Step 7.1: Calculate the rotation speed ($M_{rps}$) and number of pulses ($M_{n}$). Unlike steps 4 and 5, only the speed of one wheel and number of pulses required must be considered in this step. Notably, in the online phase, only the directions of rotation of the left and right wheels are different.
  o Step 7.2: Consider different *t* values to calculate $M_{d}$. According to the actual motor test, the clock frequency of the Raspberry Pi indirectly affects the pulse frequency. In other words, the generation time of each pulse is not fixed. We calculate the delay under different *t* within a range of 10–100 ms in intervals of 10 ms to improve the robustness of the dataset, *t* is randomly generated ten times within the range, and $M_{d}$ is calculated.
  o Step 7.3: Output polynomial coefficients (α, β, γ, δ). Determine the data lists of A and B for the wheel speed ($W_{v}$) and time delay ($M_{d}$), respectively, and perform polynomial equation fitting. In this process, $W_{v}$ and $M_{d}$ are the input and output values, respectively. Obtain the polynomial coefficients.
- Step 8: Calculate the time delay according to the polynomial regression formula.
- Step 9: Drive the stepping motor. According to the calculated parameter values, the Raspberry Pi board will use PWM software to simultaneously drive the left and right stepping motors to ensure that Owlbot reaches the specified speed.

**Algorithm 1:** Proposed stepping motor control algorithm**Iutput:** $V_{x}$$,\;V_{\theta }$**Output:** ${LW}_{v}$$,\;{RW}_{v}$$,{LM}_{rps}$$,{RM}_{rps}$$,{LM}_{n}$$,{RM}_{n}$$,{LM}_{d}$$,{RM}_{d}$1.  Initialize the WiringPi interface for the *LM* and *RM*2.  Declare the variables of *LW* and *RW*: ${LW}_{v}\leftarrow 0$$,\;{RW}_{v}\leftarrow 0$$,\;{LW}_{dir}\leftarrow 0$$,\;{RW}_{dir}\leftarrow 0$.3.  Declare the variables of *LM* and *RM*: ${LM}_{angle}\leftarrow 1.8^{{}^{\circ}}$
$,{RM}_{angle}\leftarrow 1.8^{{}^{\circ}}$
$,{LM}_{mic}\leftarrow 4$, … ${RM}_{mic}\leftarrow 4$
$,{LM}_{rps}\leftarrow 0$
$,{RM}_{rps}\leftarrow 0$
$,{LM}_{d}\leftarrow 0$
$,{RM}_{d}\leftarrow 0$
$,{LM}_{n}\leftarrow 0$
$,{RM}_{n}\leftarrow 0$
4.  Measure $W_{spa}$
$\;\mathrm{and}\;W_{r}$
5.  Set $W_{v-max}$
$,W_{v-min}$
$,\;M_{angle}\leftarrow 1.8^{{}^{\circ}}$
$,M_{mic}\leftarrow 4$
6.  Subscribe to the ROS topic/cmd_vel and obtain $V_{x}$
$\;\mathrm{and}\;V_{\theta }$
7.  i←0
8.  **for** all/cmd_vel(*V_x_*, *V_θ_*) **do**9.    **if**
$V_{\theta }=0$
**then**10.     ${LW}_{v}\leftarrow V_{x}$
11.     ${RW}_{v}\leftarrow V_{x}$
12.   **else**13.     ${LW}_{v}\leftarrow V_{x}-V_{\theta }*W_{spa}/2$
14.     ${RW}_{v}\leftarrow V_{x}+V_{\theta }*W_{spa}/2$
15.   **end if**16.   **if**
${LW}_{v}>0\;and\;{RW}_{v}>0$
**then**17.     ${LW}_{dir}\leftarrow 1,{RW}_{dir}\leftarrow 1$
18.   **if**
${LW}_{v}>0\;and\;{RW}_{v}<0$
**then**19.     ${LW}_{dir}\leftarrow 1,{RW}_{dir}\leftarrow 0$
20.   **if**
${LW}_{v}<0\;and\;{RW}_{v}>0$
**then**21.     ${LW}_{dir}\leftarrow 0,{RW}_{dir}\leftarrow 1$
22.   **else**23.     ${LW}_{dir}\leftarrow 0,{RW}_{dir}\leftarrow 0$
24.   **end if**25.   ${LM}_{rps}\leftarrow {LW}_{v}/2\pi *W_{r}$
26.   ${RM}_{rps}\leftarrow {RW}_{v}/2\pi *W_{r}$
27.   ${LM}_{n}\leftarrow {LM}_{rps}*(360^{{}^{\circ}}/{LM}_{angle}*{LM}_{mic})$
28.   ${RM}_{n}\leftarrow {RM}_{rps}*(360^{{}^{\circ}}/{RM}_{angle}*{RM}_{mic})$
29.   **if**
i=0
**then**30.     Generate array $S\;[W_{v-min},W_{v-min}+1,\ldots W_{v-max}$
*]*
31.     **for each**
$W_{v}\in S$
**do**32.        $M_{rps}\leftarrow W_{v}/2\pi *W_{r}$
33.        $M_{n}\leftarrow M_{rps}*(360^{{}^{\circ}}/M_{angle}*M_{mic})$
34.        In the range from 10 ms to 100 ms, randomly generate value *t* 10 times35.        $M_{d}\leftarrow \left(10^{3}-t\right)/M_{n}$
36.        Insert *W_v_* into list A, and insert *M_d_* into list B37.     **end for**38.     Create a polynomial regression equation from list A and B39.     Output polynomial coefficients: α,β,γ,δ

**40.   else**
41.     ${LM}_{d}\leftarrow \alpha *{{LW}_{v}}^{3}+\beta *{{LW}_{v}}^{2}-\gamma *{LW}_{v}+\delta$
42.     ${RM}_{d}\leftarrow \alpha *{{RW}_{v}}^{3}+\beta *{{RW}_{v}}^{2}-\gamma *{RW}_{v}+\delta$
43.     Drive the stepping motor44.     i←i+1
45.   **end if**46. **end for**

## 5. Experiments

**Figure 8:**
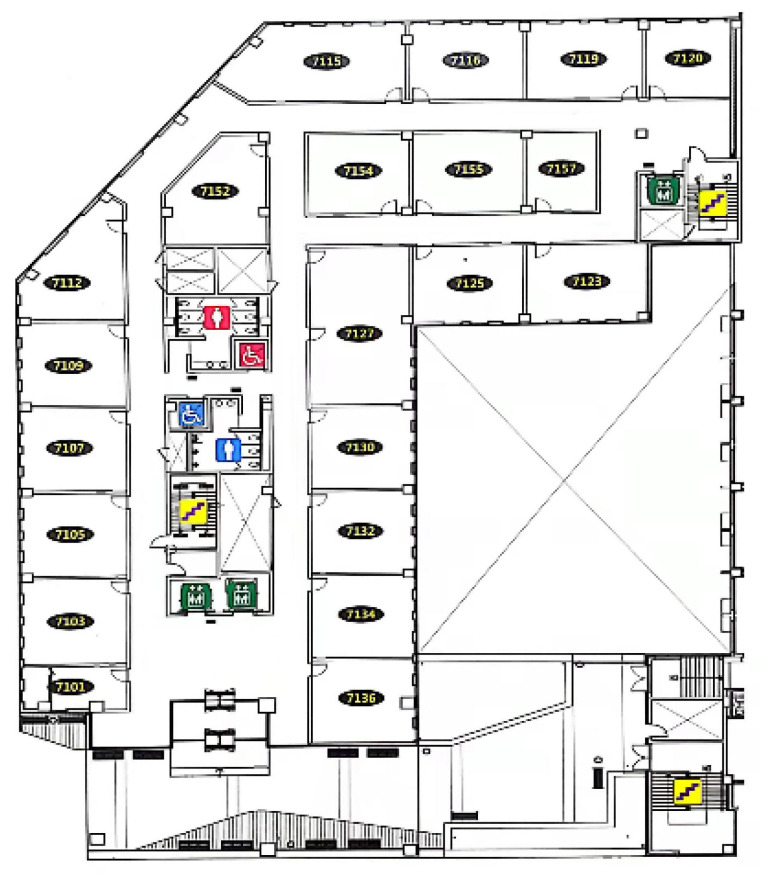
Floor plan of the experimental test scene.

Most ROS-based mobile robots can realise essential functions such as remote control movement, radar mapping, autonomous navigation, and obstacle avoidance. We assessed the crucial functionalities of Owlbot in an indoor environment. The test location was the seventh floor of the new engineering building at Dongguk University, Seoul, South Korea (Figure 8). An Alienware notebook based on the ROS melodic system was used as the robot remote control platform for the experimental configuration. The ROS master runs on a remote-controlled PC, and the remote-controlled notebook is responsible for the processing of the ROS’s main functions and information. In addition, the Owlbot and Laptop are connected to the same 2.5 GHz local area network and implement SSH communication.

### 5.1. Teleoperation Movement and Odometry Evaluation

**Figure 9:**
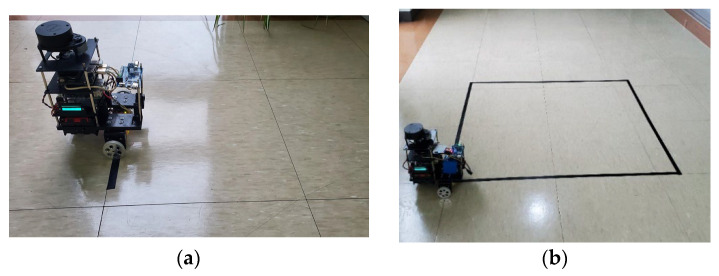
Testing scenarios. (**a**) Teleoperation movement test; (**b**) Odometer test for square line patrol.

A real-world mobile test was performed to evaluate the validity and accuracy of Owlbot’s remote control capabilities. Owlbot’s movement depends on the precise control of its stepping motors. Therefore, we designed two ROS nodes: stepping_motor_node and teleop_twist_keyboard. The stepping_motor_node program is equipped with a real-time feedback function that can display the left and right stepping motor characteristics in real time. The running parameters include the running steps, runtime (t), number of pulses (${LM}_{n}$*,* ${RM}_{n}$), and delay (${LM}_{d}$*,* ${RM}_{d}$). The teleop_twist_keyboard program can set different speed commands (cmd_vel), runtimes, and publishing frequencies. In the experiment, the publishing frequency of cmd_vel was 1 Hz. First, a uniform motion test was performed. As shown in Figure 9a, the black line was the starting point of Owlbot motion. By transmitting three sets of linear speed commands and a target runtime to Owlbot, the final moving distance of Owlbot was evaluated. Table 5 presents the twenty sets of target linear velocities with different runtime values.

In the odometry test, we sent a motion command to the robot to traverse a square path sized 1 m × 1 m. This motion command ensures that the robot turns 90° to the left after travelling 1 m at a speed of 0.05 m/s and repeats this operation four times to return to the starting point. Figure 9b shows the actual test scene. The square marked by the black line on the floor has a side length of 1 m. The topic/odom that Owlbot relies on for mobile positioning was obtained by the fusion of the nodes/odom and/Imu/data, as shown in Figure 6. The angular velocity *Z*-axis information changes when the robot moves through the four vertices in the square route. We separately recorded the data transmitted by the IMU and LiDAR and the fused odometer information.

**Figure 10:**
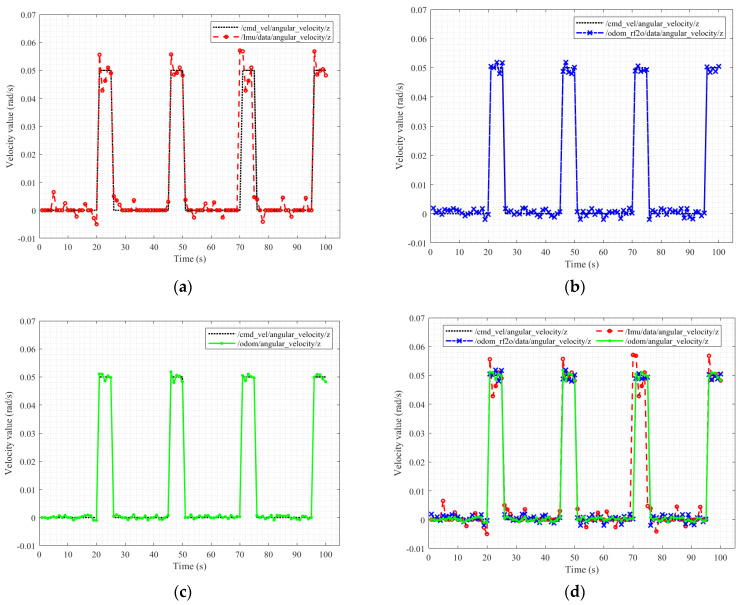
Comparison of angular velocity values of Owlbot in a walking square test. Angular *Z*-axis velocity data measured by the (**a**) IMU; (**b**) LiDAR; (**c**) IMU and LiDAR fusion. (**d**) Comparison.

As shown in Figure 10a, when the robot moves in a straight line, the angular velocity data obtained by the IMU are stable. However, when the robot turns, an error of 0.05 rad/s is observed between the measured and actual data. In contrast, the LiDAR provides stable and accurate angular velocity data when the robot turns, as shown in Figure 10b. Nevertheless, when the robot travels in a straight line, the angular velocity obtained by the radar fluctuates slightly owing to the small number of moving reference objects acquired by the LiDAR. In other words, the change in the angle of the LiDAR reflection is gradual. Figure 10c shows the results obtained by the fusion of IMU and LiDAR odometry information. For comparison, Figure 10d presents the combined results of (a), (b), and (c). The result of (c) is well matched with the speed command curve. By comparing the initial pose, LiDAR and IMU provide relative pose differences to update the EKF for pose interpretation. Specifically, the EKF estimates the pose at each moment by analysing the sensor observation data and initial pose of the initial moment. In this context, the EKF provides a more robust estimate of the robot pose than that obtained using solely LiDAR or IMU.

### 5.2. Mapping

**Figure 11:**
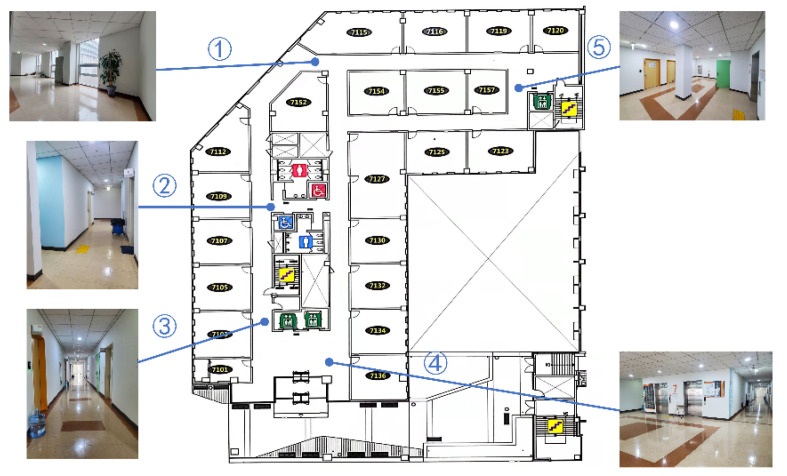
Real scene for Owlbot SLAM testing. Owlbot leaves from ①, goes through ②–⑤, and finally returns to ①.

**Figure 12:**
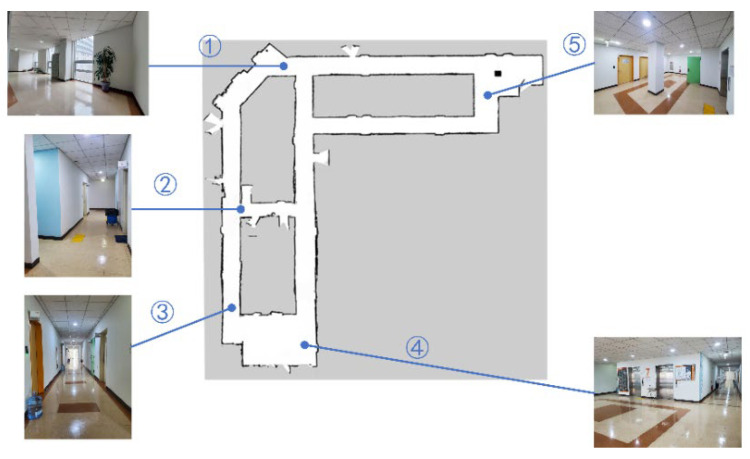
Occupation map generated by Owlbot SLAM. Owlbot leaves from ①, goes through ②–⑤, and finally returns to ①.

As mentioned, gmapping is a SLAM algorithm based on 2D LiDAR that uses the particle filter algorithm to construct 2D grid maps. This algorithm can build an indoor environment map in real time, with reasonable computations in small scenes, high map accuracy, and low requirements for the LiDAR scanning frequency. The gmapping algorithm must subscribe to/tf,/odom, and/scan to publish/map. Owlbot uses the odometry information fused with IMU and LiDAR data. Therefore, SLAM mapping can be realised using the gmapping algorithm. As shown in Figure 11, point 1 (the door of room 7115) is the starting point, and Owlbot motion through all points in the corridors is remotely controlled. LiDAR odometry data are obtained based on a cloud of points from the surrounding walls and obstacles. The platform calculates the latest changes in the translation and orientation based on the best transformation to align with the previous map. After calculating the new position and pose, the occupancy grid is updated. Figure 12 shows the SLAM maps created by gmapping. The detected obstacles and empty spaces are marked in black and grey, respectively. The map was generated by teleoperating the robot at a linear velocity lower than 0.05 m/s and an angular velocity lower than 0.5 rad/s. The constructed map was satisfactory.

### 5.3. Navigation

**Figure 13:**
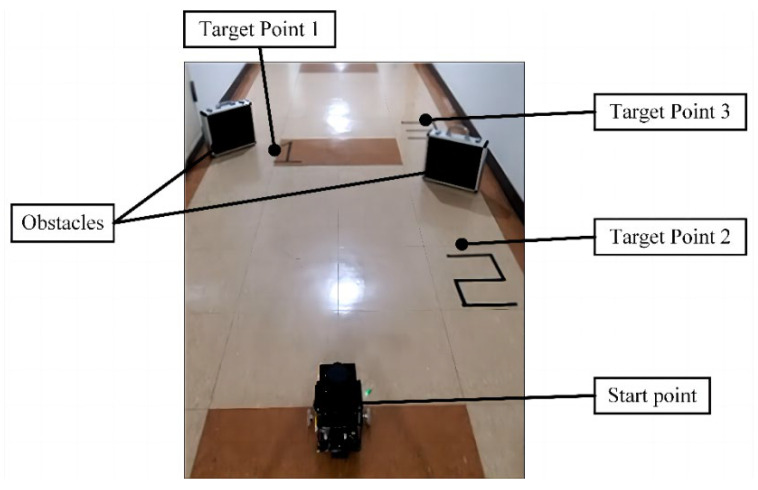
Real scene for Owlbot navigation testing.

**Figure 14:**
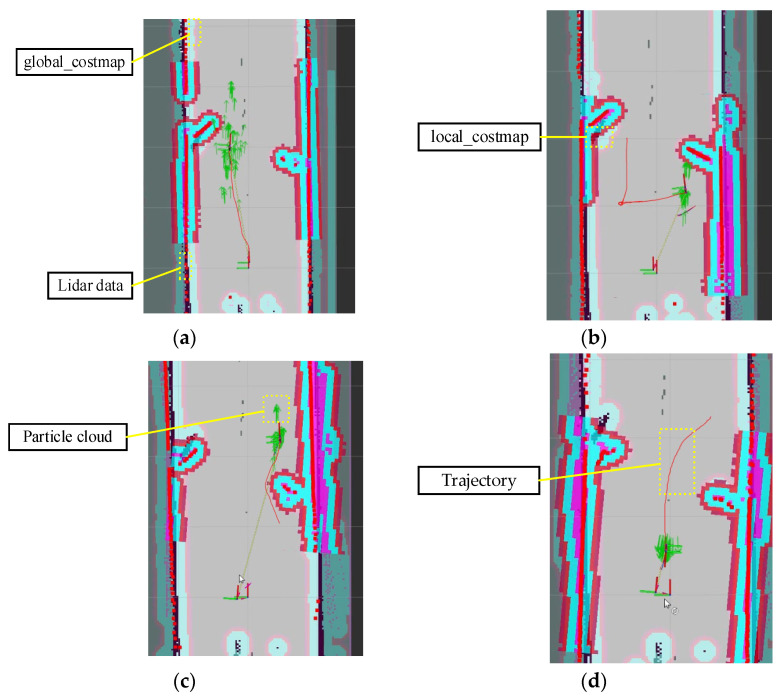
Navigation results for Owlbot, showing its movement path in (**a**) Case 1; (**b**) Case 2; (**c**) Case 3; (**d**) Case 4.

After mapping the unknown environment, Owlbot can perform precise autonomous navigation within the map range. To evaluate the navigation performance and obstacle avoidance effect of the Owlbot robot, we designed the mobile test in a corridor. As shown in Figure 13, two obstacles and three digital target points were placed on the floor. Using Rviz software, we successively issued four navigation commands: Case 1: move from the starting point to the target point 1; Case 2: move from target point 1 to target point 2; Case 3: move from target point 2 to target point 3; Case 4: return from target point 3 to the vicinity of the starting point. Owlbot uses the 2D navigation stack that inputs information from the LiDAR odometry, IMU data, and a goal pose and outputs safe speed commands that are sent to the stepping motor [39]. The navigation module of Owlbot is divided into global and local planners. The global planner finds the optimal path with prior knowledge of the environment and static obstacles by using the A-star algorithm [40]. The local planner recalculates the path to avoid dynamic obstacles by trajectory rollout and dynamic window approaches [41]. The actual navigation results are shown in Figure 14, including the global costmap, local costmap, LiDAR scan data, estimated position of the AMCL algorithm [42], and actual trajectory of the robot. In the global costmap, a light blue gradient area was generated at the edge of the obstacle, which represents the hidden cost of the robot’s possible collision with the obstacle. After the global planner obtains the navigation target point, it plans the path with the least cost in the global costmap. Owlbot advances according to the planning path. However, if an obstacle appears, the robot plans a new route through the local planner to avoid the obstacle. The local planner uses the current obstacle data obtained by LiDAR to generate a local cost map. As shown in Figure 14a, a darker colour indicates a smaller distance to the obstacle because of the higher risk. Green arrows represent the robot’s estimated position (particle cloud) derived by the AMCL. As the robot moves during the navigation process, the green arrows around it gradually converge around the robot and integrate with the real position of the robot. The solid red line represents the actual movement trajectory in the Owlbot’s navigation.

**Figure 15:**
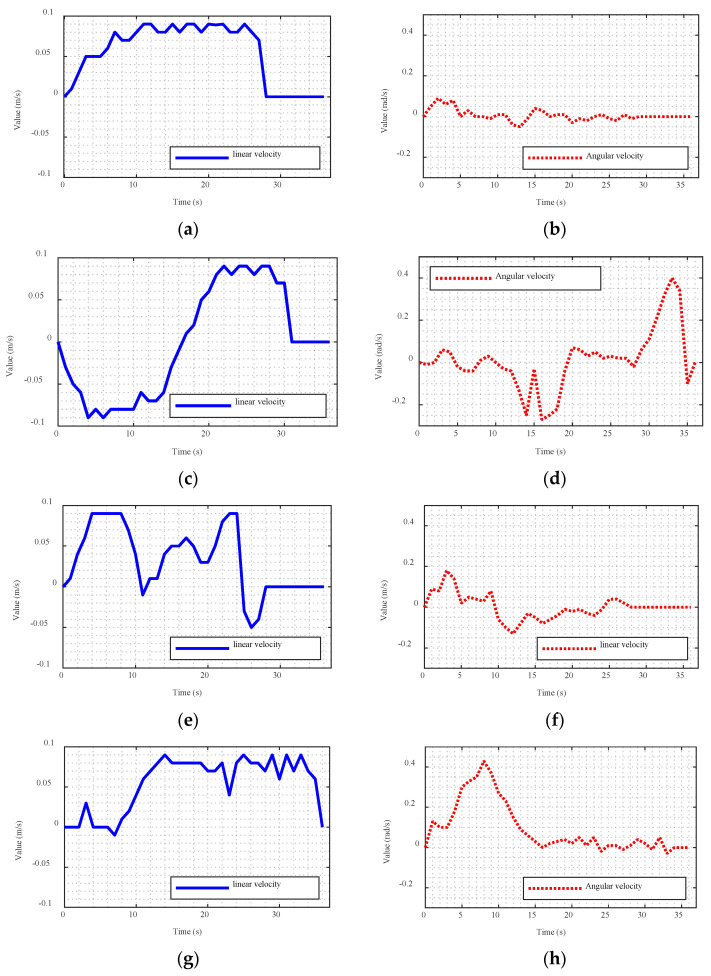
Linear and angular velocity change curves of Owlbot in four navigation tests: (**a**,**b**) Case 1; (**c**,**d**) Case 2; (**e**,**f**) Case 3; (**g**,**h**) Case 4.

Figure 15 shows the variations in the linear and angular velocities of the four cases. In Case 1, the robot slowly shifted to the left after receiving the movement command. The robot accelerated to reach target point 1 with a high linear velocity. In Case 2, the robot moved backward, i.e., the linear velocity was negative. Subsequently, the robot turned right and accelerated to the endpoint. The linear velocity (Figure 15c) decreased and then increased. Consequently, Owlbot can achieve a negative linear speed by default in the navigation configuration, corresponding to reverse driving. Owlbot can freely switch its speed and acceleration by optimising the stepping motor control algorithm. For example, in Case 3, an obstacle existed between target points 2 and 3. When Owlbot accelerated and approached the obstacle, it decelerated and passed through this area at a lower speed. Overall, Owlbot can achieve smooth speed addition and subtraction, and the movement paths are short, demonstrating its high navigation accuracy.

## 6. Conclusions

Most of the existing robot platforms have been developed based on ROS technologies. Several entry-level robot platforms are equipped with basic integrated circuit boards that can be connected to a variety of sensors. However, low-performance DC motors are typically installed as mobile actuators. The absence of encoders or other environmental sensors limits ROS-based secondary development. Robots equipped with high-performance SBC and DC motors (with encoders) can perform more advanced functions such as localisation, mapping, and navigation path planning. In this study, we designed a novel Owlbot robotic platform, which has the following advantages. First, the robot is designed to have different types of PLA chassis to carry different hardware devices or sensors, such as Jetson Nano and LiDAR. The designed power management unit can effectively supply power to the hardware devices to ensure the regular operation of the robot. Second, unlike the traditional robot platform that uses DC motors as actuators, Owlbot uses high-precision stepping motors as actuators and can achieve precise movement under the control of the proposed stepping motor control algorithm. Furthermore, the use of accurate odometry data contributes to the accuracy of SLAM and navigation. Owlbot combines 2D LiDAR and IMU information to output accurate odometer information. Overall, the Owlbot platform, which has been developed and designed based on ROS, can help researchers realise secondary algorithm development. Future work can be aimed at extending the platform to exhibit 3D SLAM functions with additional cameras.

## Figures and Tables

**Table 1:** Software, hardware, and main functions of different robot platforms.

Robot	Hardware			Software		Main Functions
	Controller/Driver	Actuators	Sensors	ROS	Programming Tool	
Mbot [24]	ATmega328	Plastic DC	UDS, LF, BT	No	Arduino IDE	OA, LP
Tiny:bit [25]	Micro:bit board	Plastic DC	UDS, LF, SS, IR	No	Graphical module/ Python	OA, LP
G1 tank [26]	Raspberry Pi/ Expansion board	DC	UDS, TR, LS, Camera	No	C/Python	OA, LP, OR
Jetbot Mini [27]	Jetson Nano/ Expansion board	Plastic DC	Camera	Yes	Python	OA, LP, OR
Jetbot [28]	Jetson Nano/ Expansion board	Plastic DC	Camera	Yes	Python	OA, FD, OR
Transbot [29]	Jetson Nano/ Expansion board	DC with OE	Depth Camera, LiDAR	Yes	Python	SLAM, Navigation
Turtlebot 3 [30]	Raspberry Pi/ Open CR	DYNAMIXELAX DC with OE	Camera, LiDAR, IMU	Yes	Python	SLAM, Navigation
Leo Rover [31]	Raspberry Pi/ LeoCore	4× DC gear motor with OE	Camera, LiDAR, IMU	Yes	Python	SLAM, Navigation
Summit-XL [32]	Intel processor/PC	4× DC gear motor with OE	Camera, LiDAR, IMU	Yes	Python	SLAM, Navigation
Owlbot	Jetson Nano/ Raspberry Pi	Stepping motor with controller	LiDAR, IMU	Yes	Python	SLAM, Navigation

Actuators: direct current gear motors (DC); optical encoder (OE). Sensors: ultrasonic distance sensor (UDS); line-following sensor (LF); Bluetooth (BT); sound sensor (SS); infrared proximity sensor (IR); tracking sensor (TR); light sensor (LS); inertial measurement unit (IMU). Functions: obstacle avoidance (OA); line patrol (LP); object recognition (OR); face detection (FD); simultaneous localization and mapping (SLAM).

**Table 2:** Owlbot components.

Items	Detail	Number
LiDAR	RPLIDAR A1	1
Single-board computer	Jetson Nano	1
	Raspberry Pi 4	1
Power management board	5 V and 12 V Output	1
Stepping motors A and B	SBC-103H548-0440	2
Motor controller	SBC-10	2
USB Wi-Fi adapter	802.11n	2
IMU	GY9250	1
Polylactide (PLA) base	3D printed	4
Wheel	Aluminium	2
Battery	Li-Po 18650	8

**Table 3:** Basic parameters of the stepping motor.

Parameter	Value
Working voltage	9–16 V
Rated voltage	12 V
Step angle	1.8°/step
Holding torque	0.265 Nm
Current	1.2 A (A/Phase)
Inductance	4.3 mH
Weight	280 g
Controller	SBC-10

**Table 4:** Parameter variables of the stepping motor control algorithm.

Variables	Parameter Description
LM, RM	left and right motors
LW, RW	left and right wheel
$V_{x}$, $V_{\theta }$	linear and angular velocities of the topic/cmd_vel.
${LW}_{v}$, ${RW}_{v}$	velocity of the left and right wheels
${LW}_{dir}$, ${RW}_{dir}$	direction of the left and right wheels
$W_{spa}$	spacing between the two wheels
$W_{r}$	wheel radius
${LM}_{angle}$, ${RM}_{angle}$	step angle of the left and right motors
${LM}_{mic}$, ${RM}_{mic}$	microstepping of the left and right motors
${LM}_{rps}$, ${RM}_{rps}$	left and right motor rotation speed (revolutions per second)
${LM}_{n}$, ${RM}_{n}$	number of pulses for the left and right motors
${LM}_{d}$, ${RM}_{d}$	delay of left and right motors
$W_{v}$	linear velocity of the wheel in the offline phase
$W_{v-max}$,$W_{v-min}$	maximum and minimum values of $W_{v}$

**Table 5:** Movement distances and distance errors for different speed commands.

Command	Linear Velocity (m/s)	Runtime (s)	Actual Movement Distance (m)	Distance Error (m)	Left Motor		Right Motor	
					${LM}_{n}$	${LM}_{d}$ (μs)	${RM}_{n}$	${RM}_{d}$ (μs)
1	0.09	1	0.088	0.002	429	2250	429	2250
2	0.026	1	0.026	0	127	7800	127	7800
3	0.068	1	0.07	0.002	343	2800	343	2800
4	0.042	1	0.04	0.002	195	5000	195	5000
5	0.072	1	0.07	0.002	343	2800	343	2800
6	0.084	2	0.164	0.004	800	2400	800	2400
7	0.048	2	0.088	0.008	430	4500	430	4500
8	0.066	2	0.136	0.004	662	2900	662	2900
9	0.062	2	0.12	0.004	604	3200	604	3200
10	0.045	2	0.096	0.006	468	4100	468	4100
11	0.026	3	0.09	0.012	438	7874	438	7874
12	0.049	3	0.162	0.015	789	3700	789	3700
13	0.048	3	0.162	0.018	789	3700	789	3700
14	0.025	3	0.084	0.009	411	7200	411	7200
15	0.049	3	0.15	0.003	732	4000	732	4000
16	–0.046	1	–0.045	0.001	220	4400	220	4400
17	–0.066	1	–0.068	0.002	331	2900	331	2900
18	–0.078	1	–0.075	0.003	365	2600	365	2600
19	–0.072	1	–0.07	0.002	349	2800	349	2800
20	–0.064	1	–0.066	0.002	322	3000	322	3000

## Data Availability

Not applicable.
